# Fluorodeoxyglucose-Positron Emission Tomography in the differential diagnosis of early-onset dementia: a prospective, community-based study

**DOI:** 10.1186/1471-2377-9-41

**Published:** 2009-08-12

**Authors:** Peter K Panegyres, Jeffrey M Rogers, Michael McCarthy, Andrew Campbell, Jing Shan Wu

**Affiliations:** 1 Neurodegenerative Disorders Research, 185 York St, Subiaco WA, Australia; 2 Neurosciences Unit, Health Department of Western Australia, Perth WA, Australia; 3 Department of Nuclear Medicine, Royal Perth Hospital, Perth WA, Australia; 4 WA PET/Cyclotron Service, Sir Charles Gairdner Hospital, Perth WA, Australia

## Abstract

**Background:**

The aim of this study was to evaluate the diagnostic accuracy of positron emission tomography (PET) using F^18 ^fluorodeoxyglucose (FDG) in the differential diagnosis of early-onset Alzheimer's disease (AD) and other dementias in a community-dwelling population.

**Methods:**

A prospective sample of 102 individuals presenting consecutively to a primary care centre for examination of suspected early-onset dementing diseases. The mean age of symptom onset of dementia in our patients was 60.06 ± 4.28 years (mean ± 1SD, 95% lower confidence intervals (CI) 54.75, upper 63.37). Patients were evaluated using standard clinical criteria for the diagnosis of dementia. Functional neuroimaging data was obtained and nuclear medicine physicians blind to the clinical diagnosis generated FDG-PET diagnoses. Final clinical diagnoses based on all available data were then established and compared against PET diagnoses.

**Results:**

Forty-nine patients received a final clinical diagnosis of early-stage AD (MMSE score 20.97 ± 5.10). There were 29 non-AD demented patients, 11 depressed patients and a miscellaneous group of 13 patients. Among patients with AD, the sensitivity and specificity of FDG-PET was 78% (95% CI: 66–90%) and 81% (95% CI: 68–86%), respectively. The positive likelihood ratio (PLR) for a FDG-PET scan positive for the diagnosis of AD was 4.11 (95% CI: 2.29–7.32) and negative likelihood ratio (NLR) for a negative FDG-PET scan in the absence of AD was 0.27 (95% CI: 0.16–0.46). The pre-test probability was 48% and post-test probability was 79.02%. The specificity of FDG-PET in the differential diagnosis of other dementias, including frontotemporal dementia, was greater than 95%.

Recruitment methods in this study provide a sample that may be more representative of patients in the general population and indicate that FDG-PET imaging can contribute to the diagnosis of AD in younger adults with major increases in the positive likelihood rates and post-test probability.

**Conclusion:**

The high specificity of FDG-PET suggests this technique might help in the diagnosis of frontotemporal dementia and other forms of early-onset dementia.

## Background

Clinical, pathologic, and genetic evidence indicates that the various dementias have different underlying aetiologies and pathogenetic mechanisms. Treatment approaches will therefore be different for each of these conditions and accurate diagnosis is critical in order to maximize the efficacy and appropriateness of specific regimes. At present, precise differential diagnosis of dementia relies on histopathological observations, only available at autopsy. Thus, when faced with a patient with a potential dementia condition, currently the clinician must establish a probable diagnosis based on evidence available from longitudinal clinical assessment, blood tests, neuropsychological evaluation, and structural brain imaging. Although in more advanced stages of dementia a pre-mortem differential diagnosis typically becomes more secure, accurate clinical diagnosis in the early stages continues to be difficult. Furthermore, with the prospect of the introduction of pharmacological therapies that might slow the rate of neurologic deterioration, dependence on the progression of disease to a more advanced stage for accurate diagnosis may subject patients to unnecessary treatment delays. Establishing valid and reliable clinical markers of dementia capable of identifying differential pathognomonic change during the early clinical stages and with young onset is required.

Early differential diagnosis and management of dementia may benefit from precise functional neuroimaging information. In disease states of the central nervous system including dementia, the ability of neurons to take up metabolites such as glucose is impaired. By identifying regions of hypometabolism, functional neuroimaging techniques, including positron emission tomography (PET), can theoretically assist in the clinical evaluation and differentiation of dementia syndromes [1-5].

### PET and Dementia

A number of PET studies have identified distinct patterns of brain metabolic abnormalities indicative of a disruption of neuronal function in individuals diagnosed with various dementia syndromes, including Alzheimer's disease (AD) [6-8]; frontotemporal dementia (FTD([9-11]; vascular dementia (VD) [12]; primary progressive aphasia (PPA) [13,14]; dementia with Lewy bodies (DLB) [15-17]; and depression [18,19]. PET studies are therefore increasingly being used as an adjunct in the clinical evaluation of patients with suspected dementia, particularly to aid in early detection [1,17,20], or when a clinical diagnosis is problematic [2,7,16,21,22]. However, the actual sensitivity and specificity of PET in the diagnosis of dementia in young adults with the onset of dementia syndrome prior to the age of 65 years is unclear.

### Diagnostic Accuracy of PET

Multiple PET studies have shown that individuals diagnosed with AD demonstrate a characteristic pattern of glucose hypometabolism, and the condition can be distinguished from healthy controls with 93–94% sensitivity and 93–99% specificity [23,24]). The capability of PET to differentiate AD from other types of dementia is more variable, with sensitivity values as high as 93–94% [21,25] but as low as 44% [1], and specificity values ranging from 63 to 80% [1,22,25].

While the majority of functional neuroimaging research has focused on identifying AD, the sensitivity and specificity of PET in diagnosing other dementia conditions has also been investigated. PET distinguished FTD from AD or DLB with 78% sensitivity and 71% specificity [3]. DLB has been differentiated from AD with 85–90% sensitivity and 80–91% specificity [3,16,26].

Unfortunately, these studies of the diagnostic accuracy of PET are fraught with a number of methodological limitations. It is therefore difficult to assess the applicability of the reported diagnostic values to routine practice [27,28].

Foremost, studies retrospectively recruited patients non-consecutively from specialty clinics, often resulting in cohorts composed entirely of individuals with manifest dementia of one specific type [6,22,23,25,26]. This method of recruitment resulted in homogeneous patient samples that are not generally representative of those individuals who undergo dementia evaluations in primary health care settings. Furthermore, when only those patients who have tested positive on clinical grounds are used to evaluate the accuracy of a diagnostic test, verification bias can occur, leading to substantial bias in the estimates of test performance [29]. To be more clinically applicable, a study should include a spectrum patients ranging from those at risk for a particular disease (such as mild cognitive impairment) to those with manifest disease [30]. Assessing only a subset limits the clinical applicability of the results. While no formal description of patient recruitment was provided, one study appeared to consecutively recruit its sample from a primary care centre [1]. Not unexpectedly, this study reported PET diagnosed AD with 44% sensitivity, a much lower value than reported in those studies with homogeneous, retrospectively selected sample populations.

Second, confidence of the results in a number of studies is limited by small numbers of patients [1,3,16,21,26]. To address the problems associated with small sample sizes, two large, multi-centred studies have been conducted [24,25]. However such a research design lacks standardization, and the lack of uniformity of procedures among contributing sites for recruiting participants, collecting clinical data, and recording and categorizing PET presents a potentially significant confound [27].

Third, the highest diagnostic values have come from studies investigating the ability of PET to distinguish dementia from healthy controls [23,24], while values generated when differentiating amongst various dementia conditions are generally lower and more variable [1,21,25]. The highest sensitivity and specificity values may not therefore generalize to clinical practice, where the clinician is often faced with the difficult task of differentiating between multiple potential conditions, rather than simply dissociating between manifest dementia and general good health.

Finally, accuracy of PET diagnosis is frequently discussed only in terms of sensitivity and specificity. Sensitivity refers to the probability of a positive test among patients with disease, while specificity refers to the probability of a negative test among patients without disease. However, clinicians don't generally know whether or not a patient has disease, hence the need for ordering the test. Thus, while sensitivity and specificity are among the most commonly reported methods for communicating the diagnostic value of a particular test, they do not convey the information needed to interpret test results. Ideally, one would like to know what the probability of disease is given a positive or negative test. For this, likelihood ratios can be calculated to assess the likelihood of disease. The positive likelihood ratio (PLR) indicates the increase in probability of disease following a positive test, while the negative likelihood ratio (NLR) represents the reduction in probability of disease following a negative test result.

### Aims of the Current Study

It is uncertain whether the diagnostic sensitivity and specificity of PET is suitably high enough to be of value in the diagnosis of dementia. The aim of the current study was therefore to evaluate the value of PET imaging in supporting the clinical diagnosis of common dementia syndromes in a sample of individuals presenting consecutively to a primary care centre for examination of suspected neurological impairment in young adults. Such a community-based case series is more representative of the type of patients presenting for dementia investigation and the results of the data analysis more appropriate for guiding diagnosis.

## Methods

### Participants

All individuals referred to a young onset dementia clinic for specialist neurologic investigation of suspected dementia over the years from 1998 to 2006 were included in the current study.

In Perth, Western Australia, patients suspected with early-onset dementia are referred to the State referral centre within the Neurosciences Unit, Department of Health, Western Australia. Referral to this clinic is open to general practitioners, neurologists, psychiatrists and other physicians; patients and their families can self refer. A total of 102 consecutive community-dwelling patients with the suspicion of early-onset dementia were entered into the study. The clinical diagnoses of AD, FTD, DLB and PPA were made using accepted criteria as previously published [31]. This patient group represents a large proportion of individuals with early-onset dementia in Perth, Western Australia, as the majority are assessed and managed in this clinic [31].

The cohort was composed of 102 consecutively presenting patients, and included 55 males and 47 females. The mean age of symptom onset of dementia in our patients was 60.06 ± 4.28 years (mean ± 1 standard deviation (SD); 95% lower CI 54.75, upper 65.34) (Table 1). Patients received a diagnosis based on standardized clinical assessment [31] utilizing widely accepted diagnostic criteria, including longitudinal clinical assessment, blood tests, neuropsychological evaluation, EEG analysis, and structural brain imaging [32-36].

**Table 1:** Characteristics of the Patient Sample

**Diagnostic Group**	**Number (men/women)**	Age at Symptom Onset*	Age at PET Scan*	**MMSE Score (max = 30)^+^**	Disease Duration (years)*
Cohort	102 (55/47)	60.06 (4.28)	64.04 (8.90)	25 (5–30)	5.74 (2.75)
AD	49 (24/25)	61.75 (9.73)	65.65 (9.41)	20.97 (5.10)	5.34 (2.08)
FTD	17 (10/7)	59.87 (6.72)	63.42 (7.80)	25.44 (3.32)	5.19 (2.27)
DLB	6 (6/0)	64.47 (5.13)	69.19 (4.65)	27.40 (1.82)	6.68 (2.85)
PPA	6 (3/3)	61.18 (8.66)	67.54 (7.59)	21.00 (10.65)	7.28 (3.37)
Depress	11 (6/5)	53.02 (8.28)	56.29 (7.92)	27.11 (2.89)	5.34 (1.85)

Note: Depress = Depression. *Values are Means (*SD*). ^+ ^Values are Medians (Range)

The diagnosis of AD was based on the McKhan et al. [33] and DSM-IV criteria[34], supported by neurological examination, structural imaging in the form of MRI (unless contraindicated in which case high resolution CT scanning was performed), and supported by neuropsychometry, blood investigations and EEG analysis. Similarly, FTD and PPA were diagnosed using the criteria of Neary et al. [35] and Mesulam [37,38], and supported by neurological assessment, structural imaging, neuropsychometry, blood analyses and EEG. DLBD using the McKeith criteria of 1996 [32] was used, supported by structural imaging and other assessment as stated above. Depression was diagnosed using DSM-IV criteria [34].

**Figure 1:**
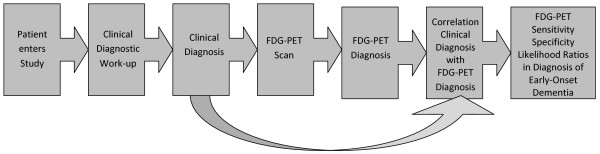
**Study Design FDG-PET in diagnosis of early-onset dementia**.

The clinical diagnostic information was not divulged prior to PET scanning. The PET scans were analysed without knowledge of the clinical diagnosis. The study design is pictorially presented in Figure 1.

The research was performed with the approval of the Ethics Committee of Graylands Selby Lemnos Hospital. The research was carried out in compliance with the Helsinki Declaration and subjects gave informed consent.

Final clinical diagnoses were: 49 patients with AD; 17 patients with FTD; 11 patients with depression; six patients with DLB; six patients with PPA; and a miscellaneous group of 13 patients (4 patients with other conditions; 2 patients with mild cognitive impairment; 2 patients with vascular dementia; 2 patients with progressive supranuclear palsy; 2 normal patients; and 1 patient with corticobasal syndrome). The results of FDG-PET were not included in the final clinical diagnoses. The PET diagnoses were then compared with the final clinical diagnoses.

Presenting complaints included cognitive decline (85.29%), speech disturbance (8.82%), behavioural change (4.90%), and gait abnormality (0.98%). Twenty-eight patients (27.45%) self-reported a positive family history of dementia.

The duration of follow-up for diagnostic classifications used in the study were: AD = 5.35 years ± 1.31 (mean ± 1 SD); FTLD = 5.39 years ± 1.29; DLB = 5.25 years ± 1.35; Depression = 5.89 years ± 1.52. The patients were seen in regular follow-up throughout the study and this information was always available.

### PET Imaging and Data Analysis

Flurodeoxyglucose (FDG) was synthesised by automated synthesis modules: IBA ^18^F-FDG Module with GE TracerLab Mx Module. All patients were imaged utilising an Allegro GSO PET scanner (Philips Medical Systems). Patients were instructed to fast for at least six hours, before FDG administration weight and height was measured and euglycaemia was confirmed. A standard patient dose adjusted for surface area was given (Dose (MBq) = 370 × BSA(patient)/1.88). All patients were imaged at rest in a quiet room with dim lights and minimal environmental stimulation. A 45-minute uptake period following FDG administration and prior to imaging was used.

Brain images were attenuation corrected using the ^137^Cs attenuation source build into the Allegro camera system. Scatter and random correction was performed as part of the RAMLA-3D reconstruction algorithm as provided by the camera manufacturer, Phillips.

The FDG PET images were reported off the Siemens work stations following reorientation and windowing using the Siemens "cool" colour scale. The intensity was adjusted such that 'normal' brain, typically cerebellum or basal ganglia was at the 100% level of the colour scale. Transaxial images were reconstructed using interactive reconstruction (3D-RAMLA) and measured transmission attenuation correction. Functional data were analysed semi-quantitatively using the Neurostat brain analysis software package. Each study was transformed using linear scaling and non-linear warping to match the Neurostat standard Talairach anatomical atlas. Maximum cortical activity was extracted using the three-dimensional stereotactic surface projection (3D-SSP) method described by Minoshima et al., [39] and the data sets were normalized to the average cerebral count for each patient.

The 3D-SSP images were compared individually with age-appropriate and modality appropriate normal databases generated in our PET centre using pixel-by-pixel 2 score analysis. A statistically significant threshold, controlling for multiple pixel comparisons and shape of the stochastic process on 3D-SSP format, of Z = 4.53 (p < 0.05) was used. The severity of the reductions in each of the lobes was evaluated using volumes of interest analysis.

**Figure 2:**
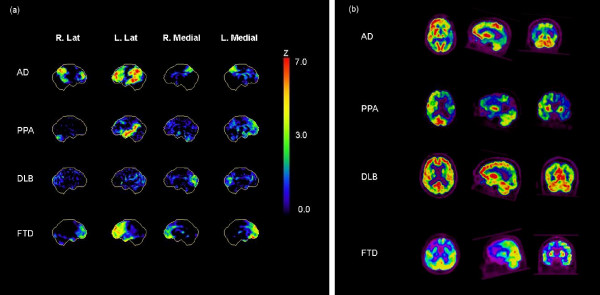
**Case examples of AD, PPA, DLB and FTD**: (a) Neurostat SSP – Z score maps, using a negative (hypoperfusion) colour scale and global normalisation; and (b) transaxial, sagital and coronal slices, centred on the area of maximal abnormality.

Normalisation of the SSP data was performed using global cortical counts. If there was the impression of extensive changes, the study was also normalised to cerebellum or pons. After database normalisation the standard deviation difference is shown as a colour scale to negative 7 standard deviations (Figure 2). The neurostat database included age and sex matched normal controls as supplied by Minoshima et al. [26].

Depending on the pattern of cerebral metabolism, each case was classified as either: normal; possible AD – biparietal hypometabolism (asymmetric changes accepted) with involvement of posterior cingulated and precuneus; possible FTLD – frontal or frontal plus temporal hypometabolism without parietal involvement – isolated temporal involvement considered possible temporal variant of FTD; possible LBD – occipital pole involvement with parietal ± frontal changes; possible PPA – predominantly dominant hemisphere superior temporal and parietal hympometabolism; or possible depression – bilateral (asymmetric changes accepted) frontal or prefrontal hypometabolism, with involvement of the anterior cingulate and basal ganglia.

Scans were reported by two experienced nuclear medicine physicians and a consensus decision was reached.

Sample sizes in each of the diagnostic groups were quite different; therefore, one-way analysis of variance could not be used to evaluate MMSE performance across groups. Rather a Kruskal-Wallis test (a non-parametric method) indicated there was no significant difference in MMSE performance between individuals diagnosed with AD, FTD, DLB, PPA or depression [F(4, 6.55) = 3.68, p = 0.07].

FDG PET data was evaluated in terms of the ability to differentially diagnose the most common dementia syndromes. Sensitivity and specificity values were calculated by comparing the blind consensus diagnosis from the PET scan against the consensus final clinical diagnosis. For all values, a 95% confidence interval (CI) was calculated according to the methods provided by Simel et al. [40].

Likelihood ratios (LR) were calculated according to the general guidelines provided by Jaeschke et al. [41]. LR is the likelihood that a given test result would be expected in a patient with the target disorder compared to the likelihood that the same result would be expected in a patient without the disorder. An LR > 1 produces a post-test probability which is higher than the pre-test probability. An LR < 1 post-test probability lower than pre-test probability. When the pre-test probability is 30–70% test results with a LR > 10 rule in disease. An LR < 1 produces a post-test proability < pre-test probability. A very low LR (eg, < 0.1) virtually rules out the chance that the patient has the disease.

## Results

### Alzheimer's Disease

Of the 49 patients who received a final clinical diagnosis of AD, the FDG-PET diagnosis correctly identified 38 of these individuals, for a sensitivity of 78% (95% CI: 66–90%). Of the 53 patients who received a final diagnosis other than AD, the PET diagnosis correctly classified 43 of these individuals as not having AD, for a specificity of 81% (95% CI: 68.86%). The positive LR for a FDG-PET scan considered consistent with AD was 4.11 (95% CI: 2.29–7.32), suggesting an increase in the likelihood of a final diagnosis of AD when diagnosed on FDG-PET with AD. The negative LR for AD was 0.27 (95% CI: 0.16–0.46), suggesting a more significant decrease in the likelihood of a final diagnosis of AD when FDG-PET findings are negative for AD. The pre-test probability prior to FDG-PET scanning that the patient had AD was 48%. Following FDG-PET scan the post-test probability was increased to 79.02% indicating that FDG-PET increases the diagnosis probability of early-onset AD from 48% to 79.02%.

### Other Dementia Syndromes

An analysis of the diagnostic accuracy of FDG-PET in other forms of dementia was also performed (Table 2). Unfortunately, the small number of patients in each diagnostic category limited the statistical confidence associated with the sensitivity results. However, these preliminary findings indicated that the specificity of PET for FTD, DLB, PPA, and depression was greater than 95%

**Table 2:** Diagnostic Accuracy of PET in other forms of Dementia

**Condition**	**N**	**Sensitivity**	**Specificity**
Frontotemporal Dementia	17	53% (29–77%)	95% (90–100%)
Dementia with Lewy Bodies	6	83% (53–100%)	99% (97–100%)
Primary Progressive Aphasia	6	50% (10–90%)	100% (99–100%)
Depression	11	18% (0–41%)	100% (99–100%)

Note: Values in parenthesis represent 95% confidence intervals

## Discussion

In a community-based series of early onset dementia, AD was detected by FDG-PET with 78% sensitivity and 81% specificity. The specificity is comparable with previously reported values, while sensitivity was slightly lower [21,25]. However, the current study group provides a sample of the general population, rather than the utilized in most previous studies. Clinicians in primary care settings can therefore take greater confidence from the findings of the current study, which demonstrates a significant increase or decrease in the likelihood of AD depending on whether PET is consistent with or not suggestive of AD.

We argue that FDG-PET is especially useful in younger patients with the suspicion of dementia. The high specificity of FDG-PET in AD, FTD and LBD implies that a negative, or normal scan, in the presence of the suspicion of dementia makes a dementia diagnosis very unlikely – in our experience excluded. In younger patients where there is a young spouse, children of teenager years or younger, the diagnostic pressure to strive for a quick and accurate diagnosis is high. Issues in relation to employment, insurance and superannuation must be dealt with. We have had experiences in our clinic where young patients (50 & 52 years) had been diagnosed with AD in other centres. This diagnosis has been called into question and after comprehensive clinical evaluation in our Centre, the diagnosis of dementia was excluded and supported by negative, or normal, FDG-PET scan. We have also seen patients (48 & 53 years) who were told by other clinicians that dementia does not occur in young adults. After a thorough clinical assessment, the patients had a profile commensurate with AD. Their FDG-PET scans were diagnostic. We strongly believe, and supported by our observations communicated here, that FDG-PET is an essential component of the diagnostic work-up of early-onset dementia. The case is less compelling for older adults (> 80 years) with the question of dementia where the impact of the diagnosis is less profound. Nevertheless, in difficult diagnostic situations we might resort to FDG-PET.

Cognitive status as measured by the MMSE was equivalent across the AD and non-AD dementia groups, and the patients were considered similar in terms of disease severity. Differential FDG-PET diagnoses therefore cannot be interpreted as simply reflecting differences in severity of disease. Rather, functional disturbances in AD appear sufficiently characteristic to allow FDG-PET to be beneficial in differential diagnosis.

With the introduction of disease modifying agents that might delay cognitive decline and maintain functional level, accurate and early diagnosis of dementia is a critical component of care. Based on performance on the MMSE, our patients with AD were considered to be in a relatively mild clinical stage. The current results clearly suggest FDG-PET can contribute in the differential diagnosis of AD in its early phases. This functional technique can therefore be recommended to not only support the diagnosis of dementia, but hasten accurate diagnosis.

Unfortunately the small number of patients in additional diagnostic categories limits the statistical confidence associated with analysis of the sensitivity and specificity of FDG-PET in identifying non-AD dementia syndromes. However, the diagnostic specificity of FDG-PET was exceptional in all the subgroups analysed. None of the 17 non-demented patients were misdiagnosed as having AD or a non-AD dementia, indicating the chance of a healthy patient being misdiagnosed with FDG-PET as having a young onset dementia syndrome is substantially unlikely. FDG-PET scanning therefore appears to be highly useful in ruling out potential dementia syndromes, and important clinical value in atypical or uncertain cases.

In the entire patient population 3.23% of all FDG-PET scans were normal or non-specific. For each diagnostic category the percentages of normal or negative scans were: 22.3% for AD (11/49); 47.0% for FTD (8/17); 33% for LBD (2/6); 50% for PPA (3/6); and 81.8% for depression (9/11). The normal or non-specific scans account for the insensitivity of FDG-PET observed in our study. There were almost no misclassifications of the FDG-PET scans as they were read independently by highly trained nuclear medicine physicians; if there was disagreement, consensus was reached. We had no situations where FDG-PET suggested a diagnosis divergent with the clinical assessment. In our experience, a normal or non-specific FDG-PET scan makes a dementing disease very unlikely.

In the current study, none of the patients diagnosed with a dementing syndrome has undergone post-mortem confirmation of diagnosis. Rather, widely accepted diagnostic criteria were used as the standard of reference for patient diagnoses. Admittedly, there is the potential for discrepancy between the clinical diagnosis and the true nature of a dementia syndrome if pathological confirmation is not obtained, and it is not possible to provide unequivocal diagnostic accuracy data. However, even pathological confirmation is not beyond reproach. There is no universally accepted set of pathological criteria, and the various diagnostic algorithms place discordant degrees of reliance on varying diagnostic factors [21]. Depending on the criteria utilized, a patient may not always receive the same autopsy diagnosis [42].

## Conclusion

Attempts to differentiate potential dementia syndromes based on clinical grounds alone can be difficult, particularly when patients present with few or an atypical profile of symptoms. In addition, clinical assessment typically involves multiple examinations and tests over months and years, which may lead to unnecessary delay in diagnosis and introduction of an appropriate treatment regime should these become available.

Little more than a decade ago, the American Academy of Neurology [43] regarded computed tomography and magnetic resonance imaging as "optional" examinations for the diagnosis and evaluation of dementia. However, structural imaging techniques have now become a widely accepted and highly valued component in the diagnosis and management of dementia [44]. A similar paradigm shift is underway with respect to the role of functional imaging, as the contribution of rapidly evolving techniques such as FDG-PET is becoming increasingly realized. The current study takes another step forward in validating the utility of functional imaging in the diagnosis of dementia, and suggests that in a clinical environment, PET may be an effective adjunct for the early diagnosis and differentiation of various dementia syndromes, especially in young adults, a conclusion supported by recent studies [45-47].

## Competing interests

The authors declare that they have no competing interests.

## Authors' contributions

PP conceived, designed, analysed, interpreted data and drafted the manuscript. JR analysed data and drafted the manuscript. JW helped analyse data; AC and MM analysed the PET data. All authors have read and approved the final manuscript.

## Pre-publication history

The pre-publication history for this paper can be accessed here:


